# Global research landscape and transfer-oriented synthesis of diet-based enteric methane mitigation in ruminants (2005–2025): A bibliometric and implementation-focused review

**DOI:** 10.14202/vetworld.2026.2067-2087

**Published:** 2026-05-18

**Authors:** Budi Wardiman, Asmuddin Natsir, Syahriani Syahrir, Aurelya Yulyanti Sudarmanto, Ahmad Rifai

**Affiliations:** 1 Department of Agricultural Science, Hasanuddin University, Makassar 90245, Indonesia; 2 Feed Chemistry Laboratory, Faculty of Animal Science, Hasanuddin University, Makassar 90245, Indonesia

**Keywords:** bibliometric analysis, diet-based mitigation, enteric methane, feed additives, greenhouse gas emissions, ruminant nutrition, transferability framework, tropical livestock systems

## Abstract

**Background and Aim:**

Enteric methane (CH_4_) emissions from ruminants are a major contributor to agricultural greenhouse gases and represent both an environmental concern and an energy loss for the animal. Despite extensive research on dietary mitigation strategies, translating experimental findings into scalable, context-specific applications remains challenging. This study aimed to map the global research landscape on diet-based enteric methane mitigation and to develop an implementation-oriented framework that links intervention efficacy to transferability across diverse production systems.

**Materials and Methods:**

A combined bibliometric and structured literature review approach was employed. A Scopus-indexed dataset (2005–2025) comprising 3,070 English-language documents from 456 sources was analyzed using the Bibliometrix package in R to evaluate publication trends, authorship patterns, country contributions, and collaboration networks. Concurrently, a structured narrative synthesis was conducted to classify dietary mitigation strategies into an implementation-relevant taxonomy and to identify constraints influencing cross-country transferability, including feeding systems, delivery mechanisms, and regulatory readiness.

**Results:**

The field exhibited rapid growth (18.38% annual growth rate), with nearly half of the publications produced since 2021. Research output involved 7,720 authors across 93 countries, reflecting strong collaboration (12.1 authors per paper; 37.72% international co-authorship) but significant concentration of influence among leading countries. The intervention landscape was organized into two principal categories: basal diet manipulation and feed additives/rumen modifiers. While numerous strategies demonstrated mitigation potential, their scalability depended largely on system compatibility, dose assurance, measurement, reporting, and verification (MRV) capacity, and regulatory frameworks rather than biological efficacy alone. Grazing-dominant and smallholder systems faced greater transfer constraints compared to confined feeding systems.

**Conclusion:**

Diet-based methane mitigation research has evolved into a rapidly expanding, collaborative field; however, practical implementation remains limited by system-specific and institutional factors. Effective mitigation at scale requires context-adapted intervention portfolios aligned with local feeding systems, infrastructure, and regulatory environments. Integrating bibliometric insights with a transferability-focused framework provides a robust basis for prioritizing research and guiding policy and industry adoption without compromising ruminant productivity.

## INTRODUCTION

Methane (CH_4_) is a critical near-term climate lever because, as a short-lived climate forcer, it exerts a disproportionately strong warming influence over the coming decades; therefore, rapid methane abatement is widely viewed as one of the fastest ways to slow the rate of warming while longer-horizon decarbonization progresses [1, 2]. International initiatives (e.g., the Global Methane Pledge) have increased demand for mitigation options that are effective, scalable, and verifiable, thereby elevating the importance of implementation-ready solutions over proof-of-concept evidence alone [3–5]. Within this agenda, ruminant livestock remain a central focus because enteric fermentation is a major anthropogenic methane source and mitigation in this domain intersects directly with food security, rural livelihoods, and the economics of animal production [6, 7]. Enteric CH_4_ also represents an energetic loss from the animal, creating a strong rationale for mitigation strategies that can align climate objectives with nutritional efficiency and productivity [8, 9]. However, adoption is ultimately determined by practicality and by the ability of interventions to fit routine feeding systems without compromising animal performance, health, or product quality [10]. In this context, the challenge is not only to identify effective strategies in principle but also to determine which strategies are feasible and transferable across diverse production environments.

Diet-based mitigation has consequently become one of the most intensively studied approaches, as it targets rumen fermentation processes and can be integrated, at least in some systems, into ration formulation and feeding management. The diet-based methane mitigation landscape can be described as spanning two broad but practically distinct categories: (i) basal diet manipulation, including changes in forage–concentrate architecture, carbohydrate fermentability, lipid inclusion through diet design, and improvements in forage and pasture management; and (ii) feed additives/rumen modifiers intended to influence methanogenic pathways more directly, such as targeted inhibitors, alternative electron acceptors, phytochemicals, and other diet-delivered bioactives. This distinction is not merely conceptual; it reflects implementation realities. Strategies that rely on controlled delivery and consistent intake are often more straightforward to deploy in confined or supplemented systems, whereas grazing-dominant contexts face intrinsic constraints related to supplementation logistics, seasonal feed variability, and limited control over intake.

Accordingly, “transferability” is considered the main analytical framework in this study and is defined as the likelihood that a diet-based methane mitigation retains meaningful methane-abatement performance when implemented across countries with different feeding platforms, delivery infrastructures, and monitoring and regulatory readiness. This challenge is conceptualized along a “knowledge-to-action continuum,” wherein uneven research geographies can hinder global scalability by shaping which intervention classes are tested under deployable conditions, standardized, and replicated beyond their original contexts. Several influential systematic and narrative reviews, as well as meta-analyses, have synthesized enteric CH_4_ mitigation options, mechanisms, and efficacy trade-offs, including portfolios of dietary strategies and rumen modifiers and quantitative syntheses of leading additives [11–17]. In parallel, recent bibliometric and text-mining studies have characterized publication trends and topical hotspots in enteric methane research; however, these studies remain largely descriptive and do not explicitly connect country-level knowledge production with an implementation-oriented intervention taxonomy [18, 19]. As a result, earlier mappings and stand-alone reviews provide limited guidance for context-specific prioritization because they do not jointly locate where evidence is produced and concentrated and organize interventions according to transferability constraints that determine scalability across heterogeneous production systems.

The absence of an integrated framework linking bibliometric knowledge distribution with implementation-oriented intervention taxonomy represents a critical research gap. Specifically, previous studies have not adequately addressed how geographical disparities in research production influence the development, validation, and deployment of diet-based methane mitigation strategies under real-world conditions. Furthermore, limited attention has been given to aligning intervention efficacy with system-specific constraints such as feeding practices, delivery mechanisms, infrastructure, and regulatory environments. This disconnect hampers the practical applicability of existing evidence and limits policymakers’ and industry stakeholders’ ability to prioritize strategies that are both effective and transferable across diverse production systems. Addressing this gap is essential to translating scientific advancements into scalable mitigation solutions that meaningfully contribute to global climate targets while maintaining livestock productivity and economic viability.

Therefore, the aim of this study was to integrate country-level bibliometric mapping with a structured, transfer-oriented literature review to develop an implementation-relevant taxonomy of diet-based methane mitigation strategies in ruminants. Specifically, this study sought to (i) analyze temporal growth patterns and thematic trends in diet-based enteric CH_4_ mitigation research from 2005 to 2025, (ii) evaluate the distribution of knowledge production and influence across countries and institutions, (iii) construct an implementation-oriented classification of dietary mitigation strategies based on their biological mechanisms and delivery requirements, and (iv) identify key transferability constraints that influence the scalability of these interventions across different production systems. By linking where evidence is produced with how mitigation strategies are operationalized, this study aims to support context-sensitive research prioritization and facilitate effective technology transfer for reducing enteric methane while maintaining livestock productivity and food-system resilience.

## MATERIALS AND METHODS

### Ethical approval

Ethical approval was not required for this study because it exclusively involved the analysis of bibliographic records and previously published literature and did not include any new experiments on animals or humans. Therefore, no live animal handling, intervention, or sampling procedures were conducted. All data were obtained from publicly accessible scientific databases and peer-reviewed publications, ensuring compliance with ethical standards for secondary data analysis and literature-based research.

### Study period and location

This study was conducted using a Scopus-indexed dataset covering the period from 2005 to 2025. The bibliographic data were retrieved and exported on 26 December 2025. The study was performed as a desk-based analytical investigation without a specific geographical experimental location; however, it incorporated global research outputs representing multiple countries and regions.

### Study design

This study used an integrated evidence-mapping approach combining (i) a country-level bibliometric analysis to describe the structure, growth, and geographic distribution of the global research landscape and (ii) a structured literature review to organize and interpret diet-based strategies for mitigating enteric methane (CH_4_) in ruminants. The bibliometric component was used to map research production, influence, and collaboration patterns, whereas the literature review component was used to develop an intervention taxonomy and to synthesize key themes and contextual considerations relevant to ruminant nutrition and cross-country transfer. These two components were integrated to link patterns of knowledge production (e.g., country output, citations, and collaboration) with the intervention landscape summarized in the review.

### Bibliometric analysis

A Scopus search was conducted to identify publications on diet-based mitigation of enteric methane in ruminants. Scopus was selected as the sole data source because it is one of the largest abstract and citation databases of peer-reviewed literature, covering more than 100 million records from approximately 28,000 active peer-reviewed journals, along with conference proceedings and books from over 7,000 publishers worldwide. This broad, multidisciplinary coverage, together with its standardized, high-quality citation metadata, makes Scopus widely used in bibliometric studies and fully compatible with bibliometric analysis tools such as the Bibliometrix package in R [20].

Records indexed were retrieved using a title, abstract, and keyword search query. The complete Scopus advanced search string was: (“enteric methane” OR “rumen methane” OR “methane emission” OR methanogenesis) AND (ruminant OR cattle OR cow OR dairy OR beef OR sheep OR goat) AND (feed OR diet OR nutrition OR “feed additive” OR “dietary manipulation”). The initial search returned 3,456 records. Filters were then applied for publication years 2005–2025, document types (articles, reviews, and conference papers), final publication stage (journal source type), and language (English), resulting in 3,070 records. These records were exported on 26 December 2025 and formed the bibliometric corpus (3,070 documents across 456 sources). Because the export date was 26 December 2025, results for 2025 should be interpreted as a partial year. Nevertheless, using a single database may introduce coverage bias, as publications indexed in other databases (e.g., Web of Science or Google Scholar) may not be fully represented; this limitation should therefore be considered when interpreting the results.

Bibliometric analyses were conducted using the Bibliometrix package in R [21]. Descriptive indicators, including annual production and growth, document types, and authorship and collaboration metrics, were computed. Country-level production was mapped based on author affiliations using a full-counting approach rather than fractional counting to reflect country participation and visibility, as each multi-country publication contributes to the knowledge base of all participating countries and this approach is commonly used in collaboration mapping. For multi-country affiliations, each country appearing in the affiliation metadata was counted only once per document; therefore, the number of country occurrences could exceed the number of documents. Country-level influence was assessed using total citations attributed to country appearances as reported in the Scopus export at the cut-off date; self-citations were not removed, and citations were not normalized by publication year. Citation totals were interpreted descriptively, acknowledging that raw citation counts favor older publications and limit cross-temporal comparability. Collaboration structure was evaluated using the proportion of internationally co-authored papers and by summarizing co-authorship networks (e.g., major hubs and cross-border links) derived from affiliation metadata.

### Structured literature review

**Scope and inclusion logic:** The literature review focused on diet-based approaches intended to reduce enteric CH_4_ in ruminants, including basal ration strategies (e.g., forage–concentrate balance, carbohydrate profile, forage quality improvement, and feeding strategy) and diet-delivered modifiers (e.g., lipid supplementation, plant bioactives, electron sinks, direct inhibitors, direct-fed microbials, and emerging materials). The primary evidence base was drawn from the Scopus corpus and, where appropriate, supplemented by high-quality, recent review and guidance papers to support the interpretation of intervention classes and implementation considerations.

Study selection and use of evidence: The bibliometric corpus was used as the starting universe, and a structured, purpose-driven selection of publications was performed to (i) define and refine the intervention taxonomy and (ii) summarize major themes relevant to ruminant nutrition practice and deployment across production systems. Review papers were used to contextualize and triangulate findings, whereas primary in vivo studies were used to exemplify intervention categories and reporting practices.

**Data charting and organization:** For papers included in the structured review synthesis, information was charted using a standardized template to improve consistency across heterogeneous nutrition studies. Charted items included animal characteristics (species or class and production stage), production system (confined or total mixed ration systems versus grazing or supplemented grazing systems), intervention type (coded using the taxonomy, including stacked or combined strategies where described), key diet descriptors (basal diet type and major nutrient proxies when available), methane outcome reporting format (absolute emissions, yield, and/or intensity), co-reported performance variables (e.g., dry matter intake, milk yield or composition, growth, and digestibility), and methane measurement approach (e.g., respiration chambers, sulfur hexafluoride tracer technique, GreenFeed system, or other reported methods).

**Synthesis approach:** A structured narrative synthesis was conducted and organized by intervention class and production system, emphasizing how interventions are operationalized and reported and identifying contextual factors commonly discussed as influencing applicability (diet architecture, delivery platform, animal class, and measurement method). No pooled effect estimates were produced.

**Integration of bibliometric and review findings:** Findings from the structured literature review were integrated with the bibliometric mapping by linking evidence production and amplification (country output, citation influence, collaboration, and institutional hubs) to the intervention categories dominating the published literature and to how these categories align with different production systems. This integrated interpretation was used to highlight research concentration, thematic priorities, and potential gaps relevant to cross-country learning and transfer.

## RESULTS

### Dataset overview and descriptive indicators

Drawing on a Scopus-indexed corpus spanning 2005–2025, the bibliometric dataset presented in Table 1 comprises 3,070 documents published across 456 sources. The knowledge base is overwhelmingly oriented toward primary research (85.80% articles, 9.97% reviews, and 4.23% conference papers). Authorship patterns further underline the scale of collaboration in the corpus: the dataset includes 7,720 authors, exhibits no single-authored documents, and averages 12.1 co-authors per paper, with 37.72% international co-authorship.

**Table 1:** Summary statistics of the bibliometric dataset (Scopus) used for mapping diet-based enteric methane mitigation research in ruminants, 2005–2025.

Description	Results
Timespan	2005–2025
Sources (journals, books, etc.)	456
Documents	3,070
Annual growth rate (%)	18.38
Document average age	5.81
Average citations per document	32.07
References	0
Keywords Plus (ID)	8,187
Author’s keywords (DE)	4,830
Authors	7,720
Authors of single-authored documents	0
Single-authored documents	0
Co-authors per document	12.1
International co-authorships (%)	37.72
Article	2,634
Conference paper	130
Review	306

### Annual scientific production and growth (2005–2025)

**Figure 1:**
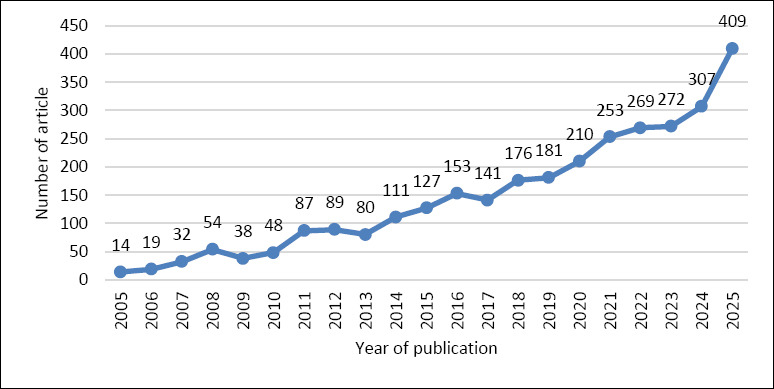
Annual scientific production in the diet-based enteric methane mitigation literature (Scopus, 2005–2025).

Across the 2005–2025 time window captured in the Scopus-derived corpus, annual scientific output on diet- and nutrition-mediated enteric methane mitigation, as shown in Figure 1, demonstrates a clear near-exponential expansion, from 14 papers (2005) to 409 papers (2025), equivalent to a compound annual growth rate of 18.4%. Importantly, this growth is not uniform but occurs in distinct acceleration phases: an early incubation phase (2005–2010; 205 papers; 6.7% of the corpus), a broadening phase (2011–2015; 494 papers; 16.1%), a scale-up phase (2016–2020; 861 papers; 28.0%), and a recent surge (2021–2025; 1,510 papers; 49.2%).

### Country-level publication output and citation concentration

**Figure 2:**
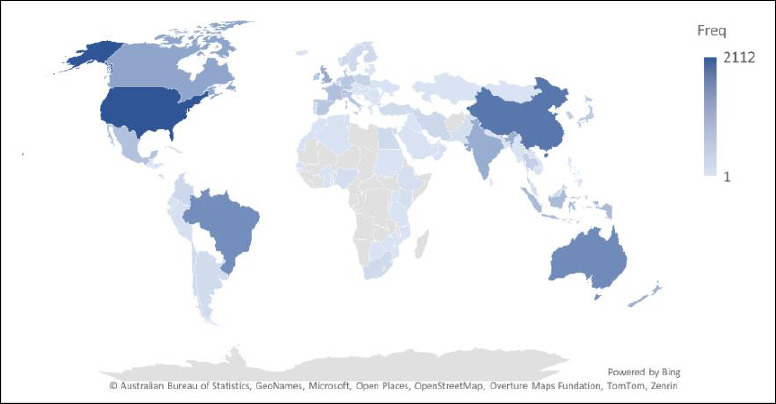
Global distribution of country contributions to diet-based enteric methane mitigation research in ruminants (Scopus, 2005–2025).

**Figure 3:**
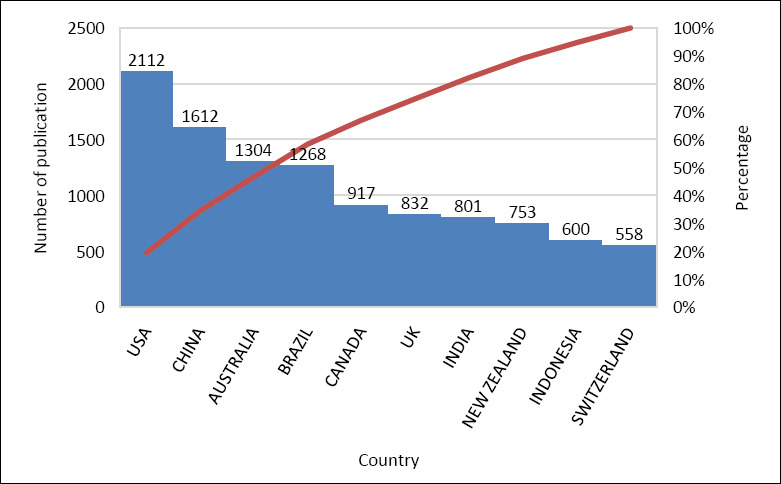
Top 10 countries with the highest publication output in the diet-based enteric methane mitigation literature (Scopus, 2005–2025).

The Scopus-based bibliometric profile indicates that research on diet-based mitigation of enteric methane is geographically broad but structurally concentrated. Across 3,070 documents, authorship affiliations span 93 countries (Figure 2), yet the top 10 countries account for 58.6% of all country occurrences. The leading contributors by participation are the USA (2,112; 11.5%), China (1,612; 8.8%), Australia (1,304; 7.1%), Brazil (1,268; 6.9%), Canada (917; 5.0%), the United Kingdom (832; 4.5%), India (801; 4.4%), New Zealand (753; 4.1%), Indonesia (600; 3.3%), and Switzerland (558; 3.0%) (Figure 3).

However, influence, proxied by country-level citations, shows an even steeper concentration: the top 10 cited countries account for 68.2% of total citations (90,271), led by the USA (12,643; 14.0%) and Canada (10,867; 12.0%), followed by Australia (7,685; 8.5%), New Zealand (5,556; 6.2%), and the United Kingdom (5,411; 6.0%) (Table 2).

**Table 2:** Top 10 most cited countries in the diet-based enteric methane mitigation literature (Scopus, 2005–2025).

Country	Total citations	Average article citations
USA	12,643	38.50
Canada	10,867	64.70
Australia	7,685	35.90
New Zealand	5,556	52.90
United Kingdom	5,411	43.60
India	4,864	34.50
China	4,748	20.30
France	4,024	49.10
Netherlands	3,062	56.70
Spain	2,746	41.60

### International collaboration patterns and institutional concentration

The complete absence of single-authored documents, together with a high mean team size (12.1 co-authors per paper) and a substantial proportion of internationally co-authored publications (37.72%), indicates that research output is predominantly generated through multi-institutional collaboration (Table 1). Institutionally, the affiliation profile suggests a distributed but hub-oriented knowledge network.

Although 1,623 distinct affiliations appear across the corpus, the top 10 affiliations account for approximately 16% of all affiliation occurrences. The most frequently appearing institutions include Aarhus University (Denmark; 359 occurrences), AgResearch Grasslands (New Zealand; 356), Wageningen University & Research (Netherlands; 291), Lethbridge Research and Development Center (Canada; 282), and Universidade de São Paulo (Brazil; 270) (Table 3).

**Table 3:** Top 10 most-relevant affiliations in the diet-based enteric methane mitigation literature (Scopus, 2005–2025).

Affiliation	Articles
Aarhus Universitet	359
AgResearch Grasslands	356
Wageningen University and Research	291
Lethbridge Research and Development Center	282
Universidade de São Paulo	270
Pennsylvania State University	229
Université Clermont Auvergne	190
Khon Kaen University	187
Universidade Estadual Paulista “Júlio de Mesquita Filho”	186
Empresa Brasileira de Pesquisa Agropecuária (Embrapa)	185

## DISCUSSION

### Bibliometric growth and knowledge concentration

The novel contribution of this study is that it treats this fast-growing literature not only as an expanding body of knowledge but also as a globally distributed innovation system whose outputs must ultimately be deployable across heterogeneous production contexts. At the country level, participation was broad, with 93 countries represented in author affiliations. However, the distribution of contributions and influence was uneven: based on a full count of country appearances, the 10 most productive countries accounted for 58.64% of all country occurrences, whereas the 10 most cited countries accounted for 68.25% of total citations. This imbalance highlights why research volume and research influence should not be assumed to coincide. Building on this empirically grounded map, the integrated design of this study explicitly links where knowledge is produced and amplified with what the evidence supports. This coupling enables a more decision-relevant synthesis than either method alone, creating a transparent bridge from global knowledge production to actionable learning while preventing the common interpretive error of equating publication dominance with intervention readiness or transfer potential.

A plausible macro-level driver of the post-2020 inflection is the reframing of methane as a near-term climate lever with unusually high policy salience. This “methane moment” is reinforced by broader analyses showing that, despite rising attention, methane governance remains underdeveloped: a systematic review reported that only a small fraction of global methane emissions are covered by explicit mitigation policies, and limited measurement and verification capacity makes policy effectiveness difficult to judge, effectively creating demand for credible, quantifiable mitigation evidence, including in agriculture [3]. Within livestock scholarship, the Global Methane Pledge’s headline goal of a 30% reduction by 2030 from 2020 levels is directly referenced in recent technical reviews of enteric mitigation interventions, thereby aligning research agendas with internationally visible targets and intensifying funding and publication incentives [12].

At the discipline level, publication growth also reflects a transition toward technology and implementation readiness within ruminant nutrition research, which naturally generates more studies, syntheses, and methodological papers. First, the literature increasingly concentrates on interventions that justify regulatory-grade evidence, meta-analytic updating, and long-duration validation rather than short proof-of-concept experiments. In parallel, the accumulation of controlled trials has enabled updated quantitative syntheses for specific inhibitors, including meta-analytic work on 3-NOP, and has stimulated longer-duration studies designed to test persistence, performance implications, and practicality under production-relevant conditions, including year-long dairy supplementation and commercial feedlot-scale trials [13]. Second, the growth curve likely reflects the formalization of coordinated research funding and consortia, which accelerates output by aligning multi-institutional work around shared measurement standards and deployment questions. A review describing the Greener Cattle Initiative, for example, outlines a structured, cross-sector funding program spanning nutrition, microbiome, genetics, sensing and data technologies, and socioeconomic research, which collectively expand publication volume as projects mature [14]. Third, the post-2020 acceleration coincides with intensified attention to measurement harmonization and MRV readiness, as policy uptake and carbon accounting depend on reliable quantification. Recent technical reviews have explicitly focused on comparing and integrating methane measurement approaches, including chambers, tracer methods, and on-farm systems, to facilitate cross-study comparability [15]. Finally, a hallmark of disciplinary consolidation is the emergence of standard-setting outputs: in 2025, the Journal of Dairy Science published a dedicated special issue introduction positioning technical guidelines for the development and testing of methane-mitigating feed additives, indicating that the field is now codifying “how to do it” rather than only “what might work” [22].

Taken together, the time-series pattern in the bibliometric dataset is consistent with a field experiencing strong external climate-policy demand for methane mitigation, increasing scientific maturity of diet-based methane mitigations that warrant long-duration and regulatory-relevant evaluation, and expanding infrastructure for funding, measurement standardization, and technical guidance.

### Production system bias and transfer relevance

The production system imprint is reinforced by how high-impact nutritional reviews frame the mitigation solution space. Hristov [16] organized diet-based methane mitigation practices into diet manipulation and feed additives and discussed expected effect sizes and practicality in relation to contemporary characteristics of intensive dairy production, with explicit reference to the United States dairy context. Even without delving into efficacy rankings here, the framing itself is revealing: the field’s dominant narrative is shaped by systems in which ration formulation and additive delivery are feasible at scale, which tends to align with higher-income, technically intensive dairy and beef production environments. This does not invalidate the science; instead, it clarifies that global evidence is often built from system-specific experimental foundations and that country leadership in citations partly reflects which systems have historically generated the most generalizable experimental archetypes.

At the same time, the strong participation of Brazil, China, India, and Indonesia in publication volume indicates substantial and growing research investment in contexts that are often more heterogeneous, ranging from mixed crop–livestock systems to smallholder and tropical forage-based feeding systems. The lower citation intensity for some of these countries is consistent with, but does not prove, a research portfolio that may be more locally adapted, including region-specific feed resources, management constraints, breeds, and seasonal forage dynamics. Such adaptation can reduce immediate one-size-fits-all citability in the international literature. This interpretation aligns with recent technical perspectives emphasizing that mitigation effectiveness is expected to vary across breeds, life stages, production levels, and feeding systems and that knowledge gaps remain particularly pronounced for grazing systems [23]. In other words, countries where extensive grazing or highly variable diets dominate may generate findings that are essential for on-the-ground relevance but harder to integrate into the heavily cited, additive- and confinement-oriented core of the literature unless harmonized measurement and reporting practices expand.

System dependence is also embedded in major quantitative syntheses. Arndt et al. [17] classified mitigation strategies and evaluated their relevance across feedlot, mixed, and grassland systems, and estimated mitigation implications for different country income groupings and regional contexts. The methodological choice to stratify by production system provides a strong signal: cross-country comparisons are meaningful only when interpreted through the lens of the dominant feeding systems that underpin each country’s ruminant sector and research portfolio. Thus, the bibliometric geography observed here is best read as a coupled map of national scientific capacity and the experimental tractability of the production systems most accessible to researchers, both of which shape what becomes highly citable knowledge in dietary methane mitigation.

### Collaboration networks and knowledge governance

Across the Scopus corpus, the research enterprise on dietary mitigation of enteric methane is structurally team-science-intensive, consistent with a field that is simultaneously experimental, analytical, and translational. The complete absence of single-authored documents, together with a high mean team size and a substantial share of internationally co-authored publications, indicates that progress is typically generated through multi-institutional and cross-border partnerships rather than isolated laboratories. This collaboration pattern is not merely sociological; it reflects technical dependence on specialized infrastructure and harmonized protocols, particularly for methane measurement, where methodological choices such as respiration chambers, GreenFeed, tracer-gas approaches, micrometeorological techniques, and emerging remote-sensing integrations carry non-trivial implications for comparability, uncertainty, and external validity across production environments. The continuing methodological heterogeneity and the need to match measurement technique to research purpose, including precision versus scale, confinement versus grazing, and animal-level versus herd- or area-level inference, provide a practical explanation for why research groups coalesce into interdisciplinary consortia that combine nutrition expertise with measurement and modeling competencies [15].

Institutionally, the affiliation profile suggests a distributed but hub-oriented knowledge network. Although 1,623 distinct affiliations appeared across the corpus, the top institutions repeatedly recurred as collaboration anchors, with the top 10 affiliations accounting for approximately 16% of all affiliation occurrences. This concentration is strong enough to constitute visible hubs, yet not so concentrated as to imply monopoly control of agenda-setting. The most frequently appearing affiliations indicate that the field’s institutional backbone spans multiple continents and includes both research-intensive universities and mission-driven public research organizations. When viewed alongside the average of approximately 5.1 institutional addresses per paper in the affiliation counts, the evidence points to a routine pattern of multi-site experimentation and shared analytic pipelines rather than single-farm or single-laboratory work. This architecture is highly conducive to cross-country transfer of methods but may also amplify the influence of hubs that host measurement platforms, long-term herds, or regulatory science expertise.

A key marker that this collaboration is maturing beyond co-authorship into coordinated knowledge governance is the emergence of international standard-setting efforts. A study on methane-mitigating feed additives explicitly frames itself as the first outcome of a flagship project developed within the Global Research Alliance on Agricultural Greenhouse Gases, founded in 2011 with 68 member countries, with the stated intent to accelerate feed additive development and use and to provide technical guidelines for good practice in testing and implementation, particularly to improve capability in countries facing technical limitations [22]. This guidelines-as-infrastructure orientation is complemented by the rise of reusable evidence bases, including openly described global datasets that compile hundreds of peer-reviewed experiments with standardized variables such as diet composition, methane measurement method, performance metrics, and variance estimates. These datasets are designed to enable cross-study comparison and interaction analysis, functioning as boundary objects that allow findings generated in one regulatory, climatic, or production context to be interpreted, stress-tested, and re-parameterized for others [24].

### Systematic evidence synthesis of diet-based enteric CH_4_ mitigation

A comprehensive, structured literature review was conducted, and a taxonomic coding framework was applied to organize evidence on diet-based enteric CH_4_ mitigation in ruminants (Table 4). In this review, diet-based methane mitigation was defined as any feeding practice or diet-delivered input intended to reduce enteric CH_4_ while retaining mechanistic distinctions that support interpretation and reproducibility. At the highest level, interventions were classified into two practical pillars: (1) basal diet manipulation, including forage–concentrate profile, carbohydrate and fiber architecture, forage quality and forage-system innovation, and feeding strategy; and (2) feed additives/rumen modifiers, including direct inhibitors, alternative electron acceptors or hydrogen sinks, lipid supplementation, plant secondary compounds, direct-fed microbials, and emerging materials. This two-pillar structure is consistent with how recent authoritative nutrition reviews conceptualize the mitigation landscape and reflects a key implementation distinction: basal diet strategies primarily act by shifting substrate supply and fermentation patterns, often alongside productivity responses, whereas additive-driven approaches target microbial pathways more directly and therefore depend more strongly on reliable delivery, consistent intake, and dose control, which can vary substantially across confined versus grazing-dominant systems [23, 25–27].

**Table 4:** Classification of diet-based methane mitigations in ruminants (comprehensive literature review framework).

Intervention class (code)	Representative diet/feeding strategies	Primary biological lever (mechanistic intent)	Transfer context and delivery feasibility	Transfer enablers/constraints (what must be in place)	References
Basal diet manipulation: forage–concentrate and carbohydrate profile	Adjust forage:concentrate ratio, increase starch, modify fiber (NDF, ADF), grain/concentrate processing	Shifts fermentation toward propionate and reduces hydrogen availability, alters passage rate and fermentability	High feasibility in confined/TMR and semi-confined systems, limited precision in extensive grazing	Requires feed planning, ration formulation capacity, and consistent feed supply, risk of rumen acidosis and performance trade-offs if poorly managed	[28–31]
Forage quality and forage-system innovation	High-digestibility forages, harvest timing, silage management, legumes, tanniniferous species, pasture management	Improves digestibility and performance, some bioactive forages may suppress methanogenesis	Highly relevant for grazing and mixed systems, transferable into TMR via forage quality improvement	Requires seed and forage access, agronomy support, pasture management capacity, seasonal planning, effects depend on climate and forage ecology	[32–35]
Lipid supplementation (oils, fats, oilseeds)	Vegetable oils, protected fats, oilseeds, high-lipid concentrates	Reduces methanogenesis via decreased fermentable OM, altered VFA profile, and microbial effects	Most feasible in TMR, feedlot, and dairy systems, limited in grazing without supplementation	Requires stable lipid supply chain, mixing and delivery control, constrained by cost, milk fat response, and inclusion limits	[36–39]
Alternative electron acceptors or hydrogen sinks	Nitrate salts or nitrate-containing supplements	Diverts reducing equivalents from methane production and alters rumen redox balance	Feasible in systems with controlled dosing (TMR or supplemented systems)	Requires strict adaptation, safety monitoring, advisory protocols, regulatory acceptance varies	[40–42]
Direct methanogenesis inhibitors (synthetic)	3-NOP (mg/kg DM basis)	Inhibits methyl-coenzyme M reductase pathway in methanogens, effect influenced by basal diet	Highly feasible in confined systems with dose assurance, challenging in extensive grazing	Requires consistent delivery system, product availability, regulatory approval, MRV readiness, and cost-effectiveness	[43–45]
Direct methanogenesis inhibitors (macroalgae or bromoform-containing)	*Asparagopsis* spp. products, bromoform-containing ingredients	Disrupts methanogenesis via bioactive compounds, dose- and diet-dependent	Feasible in TMR and feedlot systems, difficult in grazing systems at scale	Requires standardized production, supply chain reliability, food safety assurance, regulatory approval, scaling constraints remain	[46, 47]
Plant secondary compounds: tannins	Condensed tannin extracts, tannin-rich byproducts, tanniniferous forages	Reduces methane via decreased fiber fermentation and microbial shifts, strongly dose-dependent	Transferable in both grazing and TMR systems, but variable	Requires local availability, safe inclusion guidance, monitoring of intake and digestibility, climate influences feasibility	[48–51]
Plant secondary compounds: essential oils and phytochemical blends	Essential oil blends, plant extracts, polyphenol mixtures	Modulates rumen microbial ecology and fermentation, generally modest effects	Mostly suited to TMR or supplemented systems	Requires product stability, consistent inclusion, limited by variability, cost, and regulatory factors	[52–56]
Ionophores and protozoa-targeting modifiers	Monensin (where permitted), protozoa control strategies	Shifts fermentation toward propionate, reduces hydrogen availability, regulation-dependent	Primarily confined systems, limited transferability	Regulatory restrictions, inconsistent responses, adoption depends on jurisdiction and residue policies	[52, 57–60]
Direct-fed microbials (DFM), yeast, microbial consortia	Yeast, probiotics, bacterial DFMs, microbiome-based products	Stabilizes fermentation and redirects hydrogen, evidence variable	Feasible in TMR and supplemented systems, limited in grazing	Requires product-specific validation, quality control, consistent intake, variability limits transfer	[61–65]
Novel materials and adsorbents	Biochar, charcoal, emerging rumen modifiers	Proposed adsorption, redox, and microbiome-mediated effects, evidence emerging	Mostly experimental and confined systems, low readiness	Requires safety validation, standardization, reproducible evidence before scaling	[66–70]

NDF = Neutral detergent fiber, ADF = Acid detergent fiber, OM = Organic matter, VFA = Volatile fatty acids, TMR = Total mixed ration, 3-NOP = 3-nitrooxypropanol, MRV = Monitoring, reporting, and verification, DFM = Direct-fed microbials.

Within each pillar, the taxonomy separates intervention families when differences in active principle, dosing conventions, and typical study designs are expected to affect interpretability and transferability. For example, lipid supplementation is coded separately from general ration reformulation because mitigation responses are closely linked to inclusion level and lipid type and because lipid interventions frequently co-vary with nutritionally meaningful outcomes, including intake, milk fat, and energy balance. Likewise, nitrate and other electron-acceptor strategies are treated as a distinct class because their mitigation mechanisms depend on competition for reducing equivalents and are inseparable from adaptation and safety management, which often shape experimental protocols and field use. Direct inhibitors such as 3-NOP are distinguished from bromoform-containing macroalgae products because they differ in active compounds, evidence streams, and practical constraints for scaling, including delivery logistics and product standardization, which are central to country transfer interpretation [46, 47, 71–73].

The taxonomy also includes an explicit category for combined or stacked strategies to preserve evidence on multi-intervention diets increasingly tested in the literature, such as an inhibitor combined with ration shifts or lipid supplementation. This is important because interaction effects, including additivity, synergy, or antagonism, cannot be inferred from single-intervention trials, and real-world mitigation packages often combine levers to achieve meaningful reductions under delivery, cost, and system constraints. Coding stacked strategies as a distinct class retains essential information about factorial structures and co-interventions that is often lost when combinations are collapsed into a single dominant ingredient, thereby improving the review’s usefulness for transfer-oriented decision-making [69, 70].

### From efficacy to deployability in country-level transfer

Diet-based methane mitigation is increasingly framed as a ready-to-scale solution, yet translating experimental efficacy into countrywide impact depends on more than biological response. The mitigation targets implied by climate pathways are large: agricultural methane reductions of approximately 11%–30% by 2030 and 24%–47% by 2050, relative to 2010 across scenarios, have been highlighted as the scale needed to remain consistent with a 1.5°C trajectory [17]. Against this backdrop, the central bottleneck becomes deployability: whether a mitigation option can be delivered reliably, verified credibly, and financed sustainably in the production realities that dominate a given country [76, 77]. In practice, even highly effective interventions can fail to deliver material national abatement if dosing cannot be ensured, regulatory approval lags, or accounting systems cannot translate animal-level reductions into farm-, supply chain-, and inventory-level reporting. This is consistent with scenario analyses indicating that, globally, only the 100% adoption of the most effective product-based and absolute strategies, including increasing feeding level and including a CH_4_ inhibitor, achieved an approximately 14% reduction by 2030. The same analysis concluded that such universal uptake is unlikely and that regions in low- and middle-income countries face additional structural constraints [77]. These findings motivate a shift in this Discussion from what works to what transfers, namely how countries can select a portfolio of dietary options that matches their feeding systems, infrastructure, regulatory environment, and MRV capacity [78].

### Transferability of dietary methane interventions

Transferability is defined here as the probability that a diet-based methane mitigation retains meaningful methane-abatement performance after moving across countries with different feed resources, production systems, and institutional readiness. Transferability, therefore hinges on an effectiveness chain: (i) biological efficacy under representative diets and animal classes; (ii) feasibility of consistent delivery and compliance at farm scale; (iii) stability of the response over time and across seasons; and (iv) the ability to account for reductions without creating leakage or upstream or downstream trade-offs that erode net climate benefit. Recent work on accounting for anti-methanogenic feed additives (AMFA) makes this distinction explicit by emphasizing that methane abatement must be assessed beyond experimental outcomes (efficacy) to include pragmatism, effectiveness, delivery methods, uncertainties, potential offsets across the life cycle, carbon-footprint tools, and even emissions trading schemes. Importantly, the appropriate accounting method must be tailored to the scale of analysis, data availability, and the intended objective, such as research inference, certification, or national inventories [25, 26, 76, 79].

### System fit as the first transfer gate

A key determinant of transferability is the feeding platform. In technical nutrition terms, diet-based mitigation options differ fundamentally in whether they require a controlled TMR or concentrate delivery, routine supplementation, or only basal diet improvements. The current evidence base remains skewed toward confined animals, and multiple high-impact reviews converge on the conclusion that considerably more work is needed to adapt anti-methanogenic strategies to grazing systems [80]. In extensive production without feed supplementation, few options are currently available, and locally applicable strategies, along with regional carbon footprint information, are repeatedly flagged as prerequisites for credible implementation. This observation is directly relevant for cross-country transfer: an intervention that scales smoothly in countries with high proportions of housed dairy cattle may be intrinsically harder to deploy in nations where ruminant production is pasture-based, fragmented, or dominated by smallholders with limited access to purchased supplements [81].

Therefore, a transfer-ready mitigation portfolio should be built hierarchically. The foundation layer includes high-transfer and lower-variance strategies such as improvements in forage quality and digestibility, strategic concentrate use, and diet balancing that can be implemented through advisory systems and feed-market development, recognizing that effect sizes may be modest and must be evaluated against productivity and profitability constraints [82]. The additive layer includes high-efficacy but system-dependent AMFA options that require consistent dosing, are often most feasible in confined or partially confined systems, and must be packaged with delivery assurance, residue, and safety evidence, and verification pathways. This framing helps prevent a common transfer error: assuming that global best equals locally feasible when the decisive constraint is frequently the delivery architecture rather than rumen biology.

### Transferability matrix for country-level adoption planning

To operationalize transferability in a way that remains useful for nutrition-focused journals and stakeholders, this study proposes an assessment matrix that countries or analysts can apply before prioritizing specific dietary options for scale (Table 5). The goal is not to replace efficacy evidence reviewed earlier but to filter interventions through feasibility, verification, and governance constraints that determine real-world abatement.

**Table 5:** Transferability assessment matrix for diet-based enteric methane interventions at the country-level.

Transferability domain	Core question for country transfer	Practical indicators	Why it matters for diet-based interventions
System alignment (feeding platform)	Does the country’s dominant feeding platform match the intervention’s delivery requirements?	Share of animals in confined/TMR, semi-confined, or grazing/extensive systems, frequency of supplementation, feed delivery infrastructure	Many high-efficacy additives require dose assurance and stable intake, grazing and extensive systems face structural limits to controlled delivery, so “best global” options may not be locally feasible

Feed-basket compatibility (local resources)	Can local feed resources support the intervention without compromising rumen function and productivity?	Typical forage types and quality, availability of concentrates or starch, lipid sources, presence of suitable carriers (minerals or premixes), baseline productivity level	Diet-based mitigation is conditioned by the basal ration, strategies effective in maize-based TMR systems may not translate directly to tropical forages or smallholder diets
Implementation capacity (delivery and services)	Can the intervention be implemented consistently under farm conditions?	Feed manufacturing or premix capacity, ration formulation and advisory services, farmer training, quality control, monitoring of intake and refusals	Transfer fails when the “last mile” is weak, even effective interventions may underperform if mixing, dosing, and compliance are inconsistent
Evidence portability (system representativeness)	Is the available evidence base generated under systems similar to the target country?	Dominant study systems in literature versus local systems (TMR vs grazing), availability of region-specific trials, similarity in diet composition and animal class	Literature is often skewed toward confined systems, evidence from dissimilar systems should be interpreted cautiously unless validated locally
MRV and decision-use fit	Can reductions be quantified and reported credibly for the intended purpose (farm, supply chain, or national inventory)?	Availability of activity data (feed, animal numbers, intake proxies), accepted emission-factor or model frameworks, feasibility of on-farm verification, alignment with national inventory methods	Transferability improves when mitigation can be measured or estimated credibly, otherwise benefits are difficult to claim, finance, or scale
Regulatory and safety readiness	Are approval and safety requirements compatible with the intervention class?	National feed additive regulations, residue and food safety requirements, restrictions on additive categories, required evidence dossiers	Regulatory differences can be a major bottleneck, especially for additives, requiring clear authorization pathways and safety protocols
Economic viability and incentive alignment	Will producers and value-chain actors adopt at meaningful scale?	Cost of additives or practices relative to production margins, access to credit, processor incentives, policy support, supply chain participation	Adoption determines real-world mitigation, without economic incentives even effective interventions may not scale

TMR = Total mixed ration, MRV = Monitoring, reporting, and verification.

### MRV and experimental standardization

A second major barrier to cross-country transfer is methodological: inconsistent measurement and reporting undermine comparability and, by extension, confidence among regulators, supply chain actors, and finance mechanisms. High-quality guidance underscores the need for rigorous standards in study design, statistical analysis, and measurement approaches and explicitly notes that long-term AMFA studies remain scarce, which is an important limitation when countries consider scaling decisions that require confidence in the persistence of effect [25]. The same guidance recognizes respiration chambers, sulfur hexafluoride tracer methods, and GreenFeed as feasible approaches for determining efficacy, but emphasizes the need for experiments representative of the production system of interest so that conclusions remain applicable and practical [25]. Complementing this, recent methodological syntheses in animal science highlight that each quantification approach has inherent limitations and that protocol variation complicates integration across studies, precisely the problem that weakens transfer claims when moving from one country context to another [15]. Consequently, transferability improves when countries and researchers converge on harmonized reporting of diet composition, delivery method, baseline performance, measurement technique, and uncertainty because these variables are the minimum set needed to translate an observed reduction into an accounting framework suitable for inventories or certification.

### Regulatory heterogeneity and evidence requirements

Even when efficacy and MRV are strong, country transfer can stall at the regulatory interface. Recent cross-jurisdictional synthesis of AMFA approval processes shows that legal procedures and evidence requirements differ across major markets, including Australia, Canada, the European Union, New Zealand, South Korea, the United Kingdom, and the United States, implying that transfer is not simply scientific but also administrative and legal [83, 84]. For a nutrition-led mitigation portfolio, this means that the technology transfer unit is often a regulatory-ready evidence package rather than a single efficacy estimate. Countries seeking rapid adoption will benefit from designing research pipelines that generate multi-purpose evidence, including methane response under representative rations, animal health and product quality endpoints, and documentation suitable for the local feed additive framework [85, 86]. This regulatory-aware approach reduces duplication, such as each country re-running near-identical trials, and accelerates responsible scaling without weakening safety or consumer-trust safeguards.

Regulatory constraints are likely to differ not only across countries but also across intervention classes and claim types, including inhibitors, nitrate-based strategies, lipid supplementation, and macroalgae. Thus, transferability should be evaluated by both the intervention class and the country context. In addition, scientific leadership and regulatory readiness do not necessarily coincide: countries or institutions with strong citation influence may dominate the generation of efficacy evidence, but this does not automatically imply equal readiness for authorization and deployment.

In summary, country contributions to methane mitigation should be evaluated not only by the strength of efficacy evidence but also by the transfer architecture that enables scaling, including system fit, delivery assurance, MRV and accounting readiness, and regulatory pathways. These elements determine whether countries can move from research leadership to measurable abatement, thereby creating mitigation strategies that are realistically adoptable and adaptable across climates, economies, and livestock production structures [76].

### Practical implications for ruminant nutrition and the livestock sector

**Integrating mitigation into ration formulation:** For nutritionists and production advisers, one of the most actionable interpretations of the reviewed evidence is that enteric methane mitigation should be formulated as an integrated ration objective alongside animal performance, health, and product quality, rather than as a single methane-additive decision. This framing is aligned with recent authoritative syntheses that classify diet-based methane mitigation into diet manipulation and feed additives, emphasizing that mitigation must be evaluated in the context of the basal ration and the production system in which it will be implemented [11]. At the practical level, this implies a ration package approach: (i) define the relevant methane outcome metrics for the decision, including absolute emissions, yield, and/or intensity; (ii) optimize the basal diet to support intake stability and productive efficiency; and (iii) only then evaluate whether an additive or targeted modifier adds incremental mitigation without compromising performance or creating unacceptable nutritional risk. The need for metric clarity is not trivial; major reviews emphasize that selecting appropriate methane metrics is essential for interpreting climate outcomes correctly and avoiding misleading comparisons across interventions or systems [87–93].

This approach also reflects the scale of mitigation required. A meta-analysis concluded that agricultural methane reductions of approximately 11%–30% by 2030, relative to 2010, are needed for consistent climate pathways and showed that the ability to meet near-term targets is highly sensitive to the adoption and effectiveness of strategies that preserve productivity [17]. For ration design, the implication is that small methane gains that erode productivity or intake stability are unlikely to scale, whereas interventions that maintain or improve output per unit of input are more compatible with both farm economics and product-based emissions goals.

**Feed additives as controlled nutrition technologies:** Where feed additives are part of a mitigation package, the most critical operational principle is dose assurance. Technical guidelines on testing AMFA make clear that credible claims require robust experimental design, appropriate methane measurement methods, and systematic monitoring of production, physiological responses, animal health, and product quality because mitigation cannot be separated from the nutritional and commercial endpoints that determine adoption [16, 25]. In practice, this translates into quality control expectations for industry deployment: stable inclusion rates, premix integrity, homogenous mixing and feed delivery, monitoring of refusals and intake depression risk, and explicit management protocols where safety considerations apply. This is particularly important for electron-sink strategies such as nitrate, where adaptation and oversight are integral to responsible use.

Equally important is recognizing that additive performance is rarely independent of the basal ration. Recent syntheses emphasize that diet-based methane mitigation options are context dependent, with effectiveness shaped by diet composition, intake patterns, and production system constraints. Therefore, industry-facing implementation should prioritize ration–additive compatibility as a routine formulation step: the additive is not simply added but evaluated within the diet architecture that determines fermentability, hydrogen balance, and animal response. This reduces the risk that methane gains observed under tightly controlled research conditions fail to materialize on farms due to differences in basal diet, feeding management, or intake stability [87, 94–98].

Monitoring, modeling, and accounting for scalable mitigation: Scaling diet-based methane mitigation increasingly depends on whether reductions can be quantified and communicated in ways that are credible for processors, retailers, and, where relevant, inventory reporting and carbon programs. A guideline suite explicitly treats this as a systems challenge through rigorous animal-level testing standards for AMFA claims, models that can predict variability in response and support farm- to regional-scale estimation, and accounting approaches that specify how mitigation should be quantified for different uses, including life cycle assessment, farm GHG tools, and inventories, while incorporating uncertainty and the possibility of offsets outside enteric methane [25, 98–102].

For implementation, this implies a minimum MRV-ready data package that nutritionists and industry partners should aim to capture even outside research trials: diet composition descriptors, additive identity and dose basis, feeding platform, animal class and production level, intake and performance metrics, and the methane estimation method used, whether direct measurement or modeling. The emphasis on harmonized design and reporting is not only academic; technical guidance explicitly positions standardized procedures as a precondition for credible claims and wider adoption [103, 104].

**Regulatory readiness and product deployment:** Even with strong efficacy and robust monitoring, adoption can be limited by regulatory heterogeneity. A review of regulatory frameworks for AMFA authorization documents that legislation and evidence requirements differ across major jurisdictions, including Australia, Canada, the European Union, New Zealand, South Korea, the United Kingdom, and the United States, underscoring that transfer of a mitigation technology often requires a regulatory-ready evidence dossier, not only a single efficacy estimate [79]. For companies and national programs, this argues for planning implementation pipelines around standardized evidence generation, including efficacy, safety, product quality, and production responses, in formats that can be adapted to local authorization pathways, thereby reducing duplication and accelerating responsible deployment.

**Adoption and supply chain incentives:** Finally, the practical ceiling on mitigation is likely to be set less by rumen biology than by adoption dynamics. A meta-analysis showed that meeting near-term global targets is mathematically possible under extreme uptake assumptions, but also highlighted that universal adoption is unlikely and that regions with rapidly growing demand face additional challenges [17]. Complementing this, a review of enteric methane mitigation emphasized that successful implementation of safe and effective strategies requires delivery mechanisms and adequate technical support, as well as buy-in across the supply chain and consumer acceptance, which extend beyond farm nutrition decisions [11]. For subnational and national rollout, the implication is straightforward: mitigation packages should be designed for operational simplicity, integrated into routine feeding systems, and paired with incentives or value-capture mechanisms that make sustained use rational for producers.

### Future research agenda

**Research designs that improve external validity and decision utility:** A central future frontier is the transition from single-intervention testing toward explicitly combination-based methane mitigation strategies. Based on the structured synthesis developed in this study, future mitigation packages will almost certainly be built from combinations of basal diet design, targeted additives, and delivery-compatible supplementation strategies rather than isolated single levers. For diet manipulation strategies, this means trials that integrate methane outcomes with nutritionally meaningful endpoints, including DMI stability, digestibility, milk composition, and growth, under realistic forage variability and seasonal feed supply. For additive-centered strategies, the key research design challenge is to move beyond tightly controlled dosing demonstrations toward delivery assurance experiments that evaluate performance under commercial mixing, feeding, and compliance conditions.

A second priority is system-stratified evidence generation. Building on the repeated finding that grazing systems are underrepresented, coordinated multi-site trials should be designed specifically for grazing-dominant and mixed systems, using harmonized reporting and measurement approaches to ensure comparable results. Because climate-relevant impact depends on achievable adoption at scale, future work should more routinely include context descriptors that enable synthesis by system, including confined systems, TMR systems, supplemented grazing, and extensive grazing, rather than assuming interchangeability of feeding platforms. At the same time, future work should more explicitly recognize that single-intervention approaches are approaching their practical limits. As already reflected in the taxonomy used in this review, interaction effects, whether additive, synergistic, or antagonistic, cannot be inferred reliably from isolated intervention trials, even though real-world mitigation packages will often combine multiple levers under delivery, cost, and system constraints.

Accordingly, strengthening the evidence on multi-intervention stacking should be elevated from a secondary priority to a primary research direction. The practical mitigation frontier is unlikely to be a single lever; rather, it will be a ration package whose net outcome depends on how basal diet architecture interacts with rumen modifiers, supplementation routines, and delivery systems. In this sense, future mitigation strategies will almost certainly be combination-based, and single-intervention approaches are nearing the limits of their explanatory and translational value for real-world deployment. Future experiments should therefore be designed explicitly to test interactions rather than treating them as incidental. Factorial designs, combination trials, and interaction-focused analyses are especially needed to determine whether mitigation effects are additive, synergistic, or antagonistic and to identify which stacked strategies remain compatible with animal performance, ration stability, and implementation feasibility.

**Measurement, modeling, and accounting as transfer infrastructure:** Progress in diet-based mitigation will increasingly be limited by the strength of the measurement-to-decision pipeline rather than by the existence of candidate interventions. Two developments define the future agenda here. First, the field needs continued convergence on harmonized methane measurement protocols and metadata reporting because protocol variability remains a barrier to integrating global evidence [15]. Second, scaling depends on models that can predict response variability and translate animal-level abatement into farm and regional estimates. A study explicitly treats modeling and accounting as core enabling components, calling for predictive models that can support inventories and farm-level assessments while capturing uncertainty and interactions with other mitigation strategies [26].

In parallel, credible uptake in markets and policy instruments requires fit-for-purpose accounting frameworks that distinguish efficacy from real-world effectiveness and incorporate potential offsets and uncertainties across scales, including animal, farm, life cycle assessment, and national inventory scales. Research programs that generate accounting-ready evidence, including standardized methane outcomes, diet descriptors, animal performance endpoints, and uncertainty reporting, will be disproportionately valuable for accelerating responsible adoption.

**Regulatory science and adoption research:** The future research agenda must explicitly include regulatory science and adoption constraints because cross-country transfer is increasingly mediated by authorization pathways and evidence dossier requirements. A review by AMFA documented substantive differences across jurisdictions, including Australia, Canada, the European Union, New Zealand, South Korea, the United Kingdom, and the United States, underscoring that transferability often hinges on generating evidence that is not only scientifically persuasive but also regulatory-compliant [25].

Adoption-focused research should therefore move upstream. Alongside efficacy trials, studies should evaluate implementation logistics, including dose assurance and supply chain reliability, producer decision drivers, and incentive compatibility. This is particularly important given global analyses showing that the scale of methane reduction required for near-term climate alignment is substantial and that impact is highly sensitive to achievable adoption of high-performing strategies [17]. Integrating nutrition, measurement, modeling, accounting, and regulatory readiness into a single research pipeline is likely to be the most effective route to translating a rapidly growing body of literature into durable, country-adaptable mitigation outcomes.

### Limitations

This study has several limitations. First, the bibliometric analysis was based on Scopus-indexed English-language literature, which may not fully capture relevant studies published in other databases, in other languages, or in gray literature. Second, the available evidence remains dominated by studies conducted in confined or relatively controlled production systems, which limits the transferability of conclusions to grazing-based, extensive, or smallholder settings. Third, the proposed country transfer framework is conceptual and interpretive, derived from integrating bibliometric analysis and structured review rather than from a validated predictive model or a quantitative readiness index. Therefore, the conclusions should be interpreted as evidence-informed and transfer-oriented, particularly in relation to policy and implementation.

## CONCLUSION

This study provides a comprehensive and integrated synthesis of diet-based enteric CH_4_ mitigation research by combining bibliometric mapping with a structured, transfer-oriented literature review. The results demonstrate a rapidly expanding and highly collaborative research field, characterized by near-exponential growth in scientific output and strong international co-authorship. Despite broad global participation, knowledge production and citation influence remain concentrated among a limited number of countries, indicating asymmetry between research capacity and global applicability. The evidence base is predominantly derived from confined or controlled production systems, with comparatively limited representation of grazing-dominant and smallholder contexts, which are critical for global-scale mitigation.

From a practical perspective, the findings emphasize that effective CH_4_ mitigation in ruminant systems should not be approached as a single-intervention strategy but rather as an integrated ration-based framework. The classification of mitigation strategies into basal diet manipulation and feed additives or rumen modifiers highlights a key implementation distinction: while basal diet strategies offer broader transferability across systems, additive-based interventions often provide higher efficacy but require controlled delivery, dose assurance, and regulatory compliance. The concept of transferability developed in this study underscores that real-world mitigation depends not only on biological efficacy but also on system fit, delivery feasibility, MRV readiness, and regulatory alignment. These factors collectively determine whether interventions can achieve measurable and scalable impact across diverse production environments.

A major strength of this study is the integration of bibliometric analysis with an implementation-oriented review framework, which allows simultaneous evaluation of where knowledge is produced and how it can be operationalized. This dual approach provides a more decision-relevant perspective compared with conventional reviews or descriptive bibliometric studies alone. In addition, the development of a structured intervention taxonomy and a transferability-focused framework provides a practical tool for policymakers, researchers, and industry stakeholders to prioritize mitigation strategies based on system-specific constraints and opportunities.

Future research should prioritize generating system-specific and context-representative evidence, particularly for grazing-based and heterogeneous production systems. Greater emphasis is needed on long-duration in vivo studies, multi-intervention (stacked) strategies, and factorial experimental designs that capture interaction effects among dietary components. In addition, advancing harmonized CH_4_ measurement protocols and developing robust modeling and accounting frameworks will be essential to improve comparability and scalability of mitigation outcomes. Integration of regulatory science, supply chain considerations, and adoption dynamics into research design will further enhance the translation of scientific findings into actionable solutions.

In conclusion, diet-based CH_4_ mitigation is a scientifically mature and rapidly evolving field with significant potential to advance climate mitigation goals. However, achieving meaningful global impact requires a shift from efficacy-focused research toward transferability-driven implementation. By linking knowledge production with practical deployment constraints, this study provides a framework for more context-sensitive research prioritization and supports the development of scalable, credible, and system-adapted mitigation strategies for ruminant production systems.

## DATA AVAILABILITY

The data generated during the study are included in the manuscript.

## AUTHORS’ CONTRIBUTIONS

BW: Conceptualization, methodology, data curation, formal analysis, visualization, interpretation of bibliometric results, and writing – original draft preparation. AN: Conceptualization, methodology, data validation, supervision, interpretation of nutritional and methane mitigation evidence, and writing – original draft preparation. SS: Methodology, supervision, validation of the review framework, interpretation of ruminant nutrition and methane mitigation findings, and critical revision of the manuscript for important intellectual content. AYS: Literature screening, validation of intervention classification and data extraction. AR: Literature screening, data checking, reference verification, preparation of tables and figures, validation of bibliometric outputs. All authors have read and approved the final version of the manuscript.
